# A Physiological Increase of Insulin in the Olfactory Bulb Decreases Detection of a Learned Aversive Odor and Abolishes Food Odor-Induced Sniffing Behavior in Rats

**DOI:** 10.1371/journal.pone.0051227

**Published:** 2012-12-14

**Authors:** Pascaline Aimé, Chloé Hegoburu, Tristan Jaillard, Cyril Degletagne, Samuel Garcia, Belkacem Messaoudi, Marc Thevenet, Anne Lorsignol, Claude Duchamp, Anne-Marie Mouly, Andrée Karyn Julliard

**Affiliations:** 1 Centre de Recherche en Neurosciences de Lyon (CRNL), Team Olfaction: From Coding to Memory, CNRS UMR 5292 - INSERM U1028- Université Lyon1 - Université de Lyon, Lyon, France; 2 Métabolisme Plasticité Mitochondrie, CNRS UMR 5241 - Université Paul Sabatier, Toulouse, France; 3 STROMALab, CNRS UMR 5273 - EFS - INSERM U1031- Université Paul Sabatier, Toulouse, France; 4 Laboratoire d'Ecologie des Hydrosystèmes Naturels et Anthropisés (LEHNA), CNRS UMR 5023 - Université Lyon 1 – Université de Lyon, Villeurbanne, France; 5 Department of Pathology and Cell Biology, Columbia University Medical Center, New York, New York, United States of America; Hosptial Infantil Universitario Niño Jesús, CIBEROBN, Spain

## Abstract

Insulin is involved in multiple regulatory mechanisms, including body weight and food intake, and plays a critical role in metabolic disorders such as obesity and diabetes. An increasing body of evidence indicates that insulin is also involved in the modulation of olfactory function. The olfactory bulb (OB) contains the highest level of insulin and insulin receptors (IRs) in the brain. However, a role for insulin in odor detection and sniffing behavior remains to be elucidated. Using a behavioral paradigm based on conditioned olfactory aversion (COA) to isoamyl-acetate odor, we demonstrated that an intracerebroventricular (ICV) injection of 14 mU insulin acutely decreased olfactory detection of fasted rats to the level observed in satiated animals. In addition, whereas fasted animals demonstrated an increase in respiratory frequency upon food odor detection, this effect was absent in fasted animals receiving a 14 mU insulin ICV injection as well as in satiated animals. In parallel, we showed that the OB and plasma insulin levels were increased in satiated rats compared to fasted rats, and that a 14 mU insulin ICV injection elevated the OB insulin level of fasted rats to that of satiated rats. We further quantified insulin receptors (IRs) distribution and showed that IRs are preferentially expressed in the caudal and lateral parts of the main OB, with the highest labeling found in the mitral cells, the main OB projection neurons. Together, these data suggest that insulin acts on the OB network to modulate olfactory processing and demonstrate that olfactory function is under the control of signals involved in energy homeostasis regulation and feeding behaviors.

## Introduction

The physiological regulation of food intake and body weight relies on endocrine signals, such as insulin, continuously informing the central nervous system (CNS) about the circulating and stored levels of nutrients in the organism. Insulin is secreted by pancreatic β-cells in response to blood glucose levels [1] and insulin levels are correlated with the percentage of body fat [2]. Insulin facilitates glucose metabolism by promoting circulating glucose uptake into peripheral tissues [3]. Additionally, insulin crosses the blood-brain barrier by a saturable transporter to reach the CNS and to regulate energy homeostasis [4], [5], [6], [7]. Third ventricular injections of insulin decrease food intake and body weight [8], [9], [10], [11], [12], [13], and the selective depletion of insulin receptors (IRs) in neurons results in hyperphagia leading to obesity [14].

An organism's feeding state influences olfactory processing. Indeed, short-term fasting increases, and satiation decreases, olfactory detection for neutral odors [15]. A 48 h starvation increases exploratory and sniffing behaviors as well as *c-fos* activation in the mitral and granular cell layers of the olfactory bulb (OB) in response to food odors [16]. In addition, OB mitral cell reactivity is increased by short-term starvation and insulin-induced hypoglycemia and decreased by re-feeding and gastric distension [17], [18], [19], [20]. Several evidences suggest that insulin within the OB might be involved in the modulation of olfactory processing by the feeding state. First, IRs are found throughout the brain, and the highest densities of IRs are located in the OB and the hypothalamus [21], [22], [23]. Prolonged starvation lowers insulin binding sites in the OB without affecting the hypothalamus [24]. Second, the transport rate of insulin in the OB is higher than into the entire brain [25], and the OB is one of the brain regions that contains the highest level of insulin [26]. Finally, it has been previously reported that repeated intranasal insulin administrations lead to the functional activation of downstream effectors of IRs in the OB and the modulation of several behaviors, including changes in olfactory discrimination [27].

The aim of the present study was to further investigate the role of insulin in the OB, in the modulation of olfactory processing. To this end, in the first part of the study, using acute administration of insulin in the lateral ventricle, we increased the level of insulin in the OB of fasted rats to the level of satiated rats, and measured the effects of this treatment on odor detection. First, we used a behavioral test to assess olfactory detection threshold using a conditioned odor aversion (COA) protocol in which the ingestion of a solution odorized with isoamyl-acetate at a given concentration, was paired with an intraperitoneal injection of lithium chloride. This paradigm induces a learned aversion towards the odorized drink. Afterwards, the rats were presented with lower concentrations of the odorized drink, to assess their ability to detect and so avoid the learned aversive odor. Second, we used a physiological test consisting in monitoring the animals' respiratory activity in response to a familiar food odor. Indeed, increase in respiratory frequency constitutes a reliable index of odor detection [28], [29], [30]. Our data show that increasing OB insulin in fasted rats to the level of physiologically satiated rats, decreases olfactory detection of a learned aversive odor and abolishes the increase in respiratory frequency observed during food odor presentation in fasted animals.

The second part of the study was aimed at identifying the specific cellular targets of insulin inside the olfactory bulb, and at gaining insight into their role on olfactory information processing. As a spatial organization of odor maps has been demonstrated in the OB [31]; [32], we precisely localized and quantified the distribution of IRs within the OB network. We observed that IRs are preferentially expressed in the caudal and lateral parts of the main OB, with the highest labeling found in the mitral cells, the main OB projection neurons.

## Materials and Methods

### Ethics Statement

All experiments and chirurgical procedures were conducted in strict accordance with the European Community Council Directive of November 24, 1986 (86/609/EEC), for the care and use of laboratory animals. The experimental protocols were approved by the Lyon1 University Ethics Committee (Direction of Veterinary Service #693870202), and care was taken at all stages to minimize stress and discomfort to the animals

### Animals

On arrival, adult male Wistar rats (Charles River) were housed individually in Plexiglas chambers at a constant temperature and relative humidity (22±0.5°C and 50±5%, respectively). Animals were acclimated to a 12 h light/12 h dark inverted cycle (lights off at 9:00 a.m.) with food and water *ad libitum* for at least 2 weeks. Before invasive procedures, deep anesthesia was ensured using an intraperitoneal (IP) injection of ketamine (Imalgene, 80 mg/kg) and xylazine (Rompun, 10 mg/kg).

### Surgery and injection procedures

Adult male Wistar rats (n = 18, 2 months, 250 g) were anesthetized and secured in a Stoelting stereotaxic frame, and a 22-gauge cannula (Plastics One) was placed into the left lateral cerebral ventricle using a previously described method [33]. Animals were allowed to recover for at least 7 days with food and water *ad libitum*. One week prior to testing, the rats were gradually habituated to a 22 h/day water and food restriction schedule where animals had access to food and water only from 1:00 to 3:00 p.m. Circadian insulin secretion has been shown to be mostly dependent on glycaemia and feeding [34]; [35]. Therefore, habituation to a single daily meal was imposed to synchronize the daily insulin secretion among the animal cohort.

**Figure 1:**
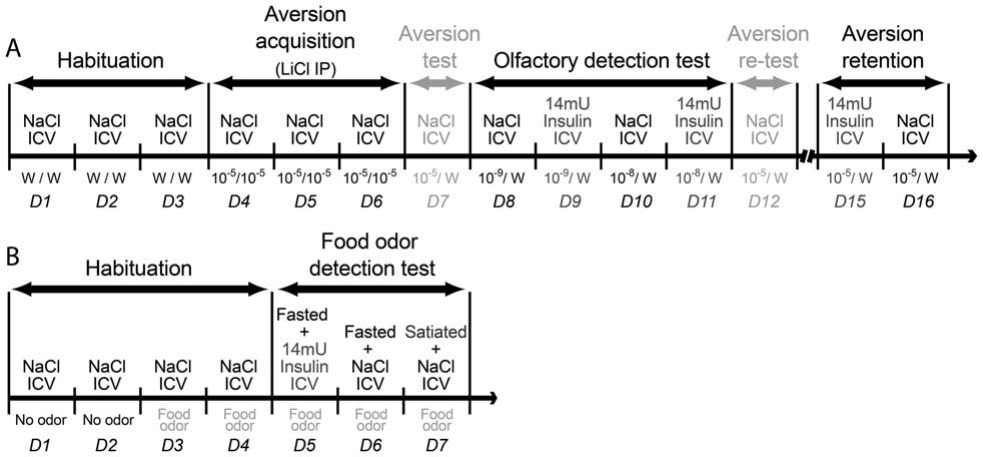
Overall course of the behavioral experiments. *(A)* Olfactory detection experiment. Animals were tested daily in the two-tube experimental device over 16 days (D1–D16). *Habituation:* On the first 3 days (D1–D3), rats were trained to drink pure water from both tubes (W/W) of the experimental cage. *Aversion acquisition:* On the following 3 days (D4–D6), rats had access to water odorized with isoamyl-acetate (ISO) diluted at 10^−5^ in both tubes (10^−5^/10^−5^). ISO 10^−5^ consumption >0.5 mL was paired with an intraperitoneal injection of LiCl (LiCl IP) to induce a conditioned olfactory aversion (COA) to ISO. *Aversion test:* On D7, the COA efficiency was tested by giving the animals a choice between water odorized with ISO 10^−5^ and pure water (10^−5^/W). On Habituation, Aversion acquisition and Aversion test days, animals were trained to receive a daily NaCl ICV injection (NaCl ICV). *Olfactory detection test:* During the Olfactory detection test period (D8–D11), rats were offered a choice between ISO 10^−9^ or ISO 10^−8^ and pure water (10^−9^/W, 10^−8^/W). For a given odorant dilution, the animals were tested on two consecutive days: once 1 h after NaCl ICV injection (D8 and D10) and once 1 h after a 14 mU insulin ICV injection (D9 and D11). *Aversion re-test:* On D12, the COA stability was assessed by giving the rats the choice between ISO 10^−5^ and pure water (10^−5^/W). *Aversion retention*: During the Aversion retention period (D15–D16), three days after the Aversion re-test, rats were offered again the choice between ISO 10^−5^ and pure water (10^−5^/W) and the animals were tested on two consecutive days: once 1 h after a 14 mU insulin ICV injection (D15) and once after a NaCl ICV injection (D16). (*B*) Sniffing experiment. Animals were tested daily in a whole-body plethysmograph over 7 days. The rats were first allowed to familiarized with the recording chamber for 4 days (Habituation), D1–D4 without (D1, D2) or with (D3, D4) food odor stimulation. During the sniffing test period (Food odor detection test, D5–D7), the animals were tested either in the fasted state (at 10:00 a.m.), 1 h after 14 mU insulin ICV injection (D5); in the fasted state, 1 h after NaCl ICV injection (D6); in the satiated state, 1 h after NaCl ICV injection (at 4:00 p.m.) (D7).

During the overall course of the two behavioral experiments (Fig. 1), 1 h before the behavioral test, rats received a daily single and unilateral intracerebroventricular (ICV injection of either NaCl (0.9%) or 14 mU insulin (human, recombinant, expressed in yeast, Sigma-Aldrich) administered in a 2 µL NaCl vehicle over 60 sec, using a 10 µL Hamilton syringe. ICV insulin injections were performed when the animals were in the fasted state (*i.e.*, when the endogenous insulin level is known to be the lowest) to ensure a maximal effect. In a set of pilot experiments, we first measured and compared OB insulin levels in fasted and satiated animals to determine the extent of the OB insulin level increase induced by a meal. This level, a 2-fold increase corresponded to the physiological OB insulin level to achieve with our ICV injection protocol. Next, we measured OB insulin levels induced by two insulin doses, 4 and 14 mU, chosen to fit within a range of insulin doses described in the literature for acute ICV injections [13], [36]. Whereas the 4 mU dose did not significantly elevate the OB insulin level (data not shown), the 14 mU dose elevated by 2-fold the OB insulin level of fasted animals, to the targeted OB insulin level of satiated animals.

### Conditioned Odor Aversion (COA) paradigm

The conditioned odor aversion (COA) protocol consisted in pairing the ingestion of an odorized drink with an intraperitoneal injection of lithium chloride, in order to induce an aversion to the odorized drink. The conditioned aversion was then tested using lower concentrations of the odorized drink, to assess olfactory detection abilities in rats ICV injected with NaCl or Insulin. Animals were tested daily in the fasted state (at 10:00 a.m.), during a 5-min session. The rats were first trained for 3 days to drink pure water at the two drinking tubes of the experimental cage (Fig. 1A: Day 1 (D1) to D3, Habituation). The experimental set-up allowed the recording of licking behavior using a two-tube device described elsewhere [15], [33]. During the next 3 days (Fig. 1A: D4 to D6, Aversion acquisition), they only had access to water odorized with isoamyl-acetate (ISO, Sigma-Aldrich). An ISO consumption of more than 0.5 mL was paired 15 min later, with an intraperitoneal injection of LiCl (10 mL/kg at 0.15 M, Sigma-Aldrich) to induce gastric malaise and establish a conditioned olfactory aversion (COA) to ISO. ISO was used at the dilution of 10^−5^, for which the solution has been shown to be tasteless [37]. Thus with this dilution, the rats can only identify the ISO solution via olfaction. Depending on individuals, one to three conditioning sessions were required for the animal to develop a strong aversion to ISO 10^−5^. On D7 (Fig. 1A: Aversion test), the COA efficiency was tested by giving the animals the choice between pure water and water odorized with ISO 10^−5^. During the Habituation, Aversion acquisition and Aversion test periods (from D1 to D7), the animals were trained daily to be gently restrained and to receive an ICV NaCl injection. Then the animals' olfactory sensitivity was assessed using lower concentrations of the ISO solution (Fig. 1A: D8 to D11, Olfactory detection test). For this, the rats were offered a choice between pure water and water odorized with ISO at lower dilutions, 10^−9^ and 10^−8^. Indeed, in a previous study, we showed that for these dilutions, the detection was modulated by the feeding state of the animal [15], [33]. Animals were tested for a given ISO dilution over two consecutive days in two experimental conditions: 1 h after NaCl ICV injection (D8 and D10) and 1 h after insulin ICV injection (D9 and D11). On D12 (Fig. 1A: Aversion re-test), the animals received an NaCl ICV injection and COA stability was assessed by giving the rats the choice between the standard ISO solution (10^−5^) and pure water. Finally, three days after the Aversion re-test, a subgroup of animals (n = 11) has been used to assess the effects of insulin ICV injection on COA retention (Aversion retention). Because extinction of a conditioned aversion is classically observed following repeated non-reinforced exposures to the conditioned stimulus [38] and could mask or overlap with the insulin effect, we tested first the effect of insulin injection, and ensured with the final control test (NaCl injection) that the conditioned aversion was still fully effective. Therefore, on D15 the rats received an ICV injection of insulin and the following day (D16), they received an ICV injection of NaCl. In both cases, 1 h later, they were given the choice between the standard ISO solution (10^−5^) and pure water. The animals' behavioral response was measured as follows. First, as previously described [33], at the beginning of each test session, the rats were intentionally placed under the tube containing the ISO-odorized water, which position was systematically changed across days. This procedure was chosen to avoid the possibility that the rats, highly motivated by thirst, would go by chance to the pure water tube first, drink only water, and not sample the ISO tube. Licking behavior was then analyzed using SciPy and MySql database software (Open Source Licenses) and an olfactory detection index was calculated, corresponding to the proportion of the number of licks at the pure water tube normalized to the total number of licks (odorized+pure water) in the experimental device during a 5 min session. When rats perceived the ISO solution (and consequently avoided it), they drank more pure water during the experimental session, resulting in a higher olfactory detection index. When two identical drinks were proposed (water for Habituation and ISO solution for Aversion acquisition periods), this index corresponded to the number of licks at the first sampled tube normalized to the total number of licks. To evaluate the animals' locomotor activity, the number of changes of side from the pure water tube to the ISO-odorized water tube during the experimental sessions was assessed and analyzed using SciPy and MySql database software (Open Source Licenses).

All percentage measures were transformed using the arcsine square root transformation to normalize the data and stabilize variance [39]. Depending on the data set, statistical comparisons were performed using either a t-test or a repeated measures ANOVA with odor dilution and ICV treatment as factors. A Student-Newman-Keuls (SNK) post-hoc test was used to complete the analysis when appropriate (Statview software).

### Respiratory activity in response to a familiar food odor

Respiratory activity during food-odor stimulation was monitored using a whole-body plethysmograph chamber (Emka Technologies, France) connected to a homemade olfactometer allowing odor delivery from the top of the chamber (for a detailed description of the experimental setup, see Hegoburu et al [30]). A subgroup of animals (n = 11) tested in COA paradigm, has been used to assess the effects of insulin ICV injection on respiratory activity in response to a familiar food odor. The rats were first familiarized to the experimental environment (plethysmograph and odor airflow) for 4 days (15 minutes per day) (Fig. 1B: D1 to D4, Habituation). The first 2 days, the animals were put in the plethysmograph chamber, with a non-odorized circulating airflow. The next 2 days, a familiar food-odor (crushed chow pellets, Harlan, France) was inserted in the airflow for 30 sec. During the test period (Fig. 1B: D5 to D7, Food odor detection test), the animals' olfactory detection was assessed by monitoring respiratory activity in response to 30 sec of food odor, in 3 different experimental conditions. On D5, the animals were tested in the fasted state (at 10:00 a.m.), 1 h after 14 mU insulin ICV injection. On D6, the animals were tested in the fasted state, 1 h after NaCl ICV injection. Finally, on D7, the animals were tested in the satiated state (at 4:00 p.m.), 1 h after NaCl ICV injection.

The respiratory signal collected from the plethysmograph was amplified and sent to an acquisition card (MC-1608FS, Measurement Computing, USA; Sampling rate = 1000 Hz) for storage and offline analysis as described elsewhere [30]. The mean respiratory frequency was calculated using SciPy and MySql database software (Open Source Licenses), as described in Hegoburu et al [30]. Four periods of ten seconds each were defined: “Pre-odor” (−10 sec to 0 sec), Odor presentation: “start” (0 sec to 10 sec), “middle” (10 sec to 20 sec), “end” (20 sec to 30 sec). The mean respiration frequency was calculated for each period in each experimental condition. A two-way repeated measures ANOVA (with period and condition as factors) was performed, followed by *post hoc* comparisons. For all the statistical comparisons performed, the significance level was set at α = 0.05.

### Physiological measurements

To evaluate physiological effects of ICV 14 mU insulin injection, we measured body weight, food consumption, glycemia, and plasma and OB insulin levels. During behavioral tests, individual body weight and food consumption were monitored daily. Statistical comparisons were performed using a paired t-test (Statview software).

We measured the effect of ICV 14 mU insulin injection on peripheral blood glucose level. For this, using the same injection schedule as for the behavioral paradigm, fasted rats received an ICV injection of either 2 µL NaCl (n = 11) or 2 µL of 14 mU insulin (n = 11) at 9:00 a.m.. Peripheral blood glucose level was determined by sampling 5 µL tail blood 1 h after ICV injections and monitoring glucose levels with a glucose meter (Accu-Chek, Roche).

We measured the effect of ICV 14 mU insulin injection on plasma and OB insulin levels and compared them to OB insulin levels in fasted and satiated animals injected with NaCl. For this, using the same injection schedule as for the behavioral paradigm, fasted rats received an ICV injection of either 2 µL of NaCl (n = 5) or 2 µL of 14 mU insulin (n = 5) at 9:00 a.m. At 4:00 p.m., satiated rats (n = 5) received a 2 µL ICV injection of NaCl. Animals were anesthetized and euthanized 1 h after ICV injections, and their OBs were immediately frozen in liquid nitrogen. Trunk blood was collected in heparinized tubes, and the plasma fraction was separated by centrifugation at 2000 g for 5 min. Insulin was extracted from OB tissues according to the procedure of Baskin et al. [26]. To determine the influence of the extraction procedure on insulin output, samples with a known amount of insulin were submitted to the same protocol. The mean extraction output was found to be ∼40%. Plasma and OB insulin levels were determined using a solid-phase, two-site enzyme immunoassay (Mercodia Ultrasensitive Rat Insulin ELISA) following the manufacturer's protocol. Statistical comparisons were performed using a non-parametric Mann-Withney test (Statview Software).

### Quantification and distribution of IRs

The effect of feeding state on mRNA, expression and distribution of IRs was evaluated. Animals habituated to a 22 h/day food restriction schedule with access to food from 1:00 to 3:00 p.m. were anesthetized and euthanized either in the fasted state at 9:00 a.m. or in the satiated state at 4:00 p.m..

We measured IR mRNA by real-time qPCR on OB of fasted (n = 6) and satiated (n = 6) rats. After rats' euthanasia, the dissected OBs were immediately frozen. Total RNA was extracted from the entire OB using Trizol (Invitrogen), and the RNA concentration and purity were assessed by measuring the optical density at 260 and 280 nm with a NanoDrop (ThermoScientific). RNA integrity was analyzed by 1% agarose gel electrophoresis (Eurobio). The relative abundance of IR mRNA was measured by real-time qPCR and normalized against β-actin. Reverse-transcription assays were performed from 1 µg of total RNA with M-MLV Reverse Transcriptase (Invitrogen). The following primer sequences were used: β-actin (sense 5′: GACGAGGCCCAGAGCAAGAGA; antisense 3′: GGGTGTTGAAGGTCTCAAACA) and IR (sense 5′-GTCTTCGAGAACGGATCGAG-3′, antisense 5′-CATGTCGGAAGAAGCAGTGA-3′). Real-time qPCR was performed with a MyiQ thermal cycler (Bio-Rad), Marnes La Coquette, France using iQ SYBR Green Supermix (Bio-Rad). The following qPCR conditions were used: 3 min at 95°C, followed by 40 cycles of denaturation for 10 sec at 95°C and annealing/extension for 1 min 15 sec at 60°C, according to the manufacturer's instructions. All samples were run in duplicate along with dilutions of known amounts of the target sequence to quantify the initial cDNA copy number (Concentration = Efficiency^ΔCt^). Results are expressed as the ratio of IR to β-actin mRNA concentrations, which was verified to exhibit non-significant variation between the different conditions. Statistical comparisons were performed using a non-parametric Mann-Withney test (Statview Software).

We measured IR expression by Western blot on OB of fasted (n = 4) and satiated (n = 4) rats. After animals' euthanasia, dissected OBs were immersed for 15 min in a hypotonic buffer (10 mM HEPES, 10 mM KCl, 240 mM sucrose and 0.5 mM dithiothreitol, pH = 7.4) supplemented with complete protease inhibitor cocktail tablets (Complete Mini, Roche). The OBs were then homogenized with a Dounce homogenizer and a B-type pestle in 60 µL of buffer. The homogenate was resuspended in 1 mL of buffer and quickly centrifuged. The supernatant (50 µL) was taken as the total fraction. Proteins (30 µg per lane) were separated on 10% SDS-PAGE gels and transferred onto a Hybond membrane (Amersham). Blocking was achieved for 1 h at room temperature in 5% non-fat dry milk prepared in Tris-buffered saline with 0.1% Tween-20 (Sigma-Aldrich). The membranes were then probed with 0.4 µg/mL of either rabbit anti-IRβ [C-19] primary antibody (#sc-711, Santa Cruz Biotechnology) or rabbit anti-IRα primary antibody (#sc-710, Santa Cruz Biotechnology) and mouse anti-actin α antibody (#MAB1501, Chemicon) overnight at 4°C. Blots were revealed using a donkey anti-rabbit (#NA934, Amersham) or goat anti-mouse (#NA931, Amersham) peroxidase-conjugated secondary antibodies depending on the primary antibody (IR subunits or actin) and an enhanced chemiluminescence kit (Amersham). Finally, the blots were exposed to autoradiographic films. An arbitrary unit of band intensity was obtained using densitometric analysis. The Western blot quantification was obtained by normalization using actin as a control housekeeping protein. Statistical comparisons were performed using a non-parametric Mann-Withney test (Statview Software).

We measured spatial distribution of IRs by quantitative immunofluorescence on OB of fasted (n = 5) and satiated (n = 5) rats. After euthanasia of the animals, the dissected OBs were immediately frozen. Immunofluorescence was performed using fresh frozen brain samples by modification of a published method [40]. Brain cryosections were preincubated for 15 min with a blocking buffer containing 0.1 M PBS (pH = 7.4), 3% BSA (Sigma-Aldrich) and 5% normal serum from the host species of the antibodies. The sections were then incubated for 2 h at room temperature with either mouse monoclonal anti-IRβ primary antibody at 4 µg/mL (1∶50, #AHR0271, Invitrogen) or mouse IgG1 as an isotype control at 4 µg/mL (1∶250, #M9269, Sigma-Aldrich) and rabbit anti-laminin (1∶100, Sigma-Aldrich) diluted in blocking buffer. The sections were washed with 0.1 M PBS/3% BSA and incubated for 1 h at room temperature with goat anti-mouse IgG conjugated with Cy3 (1∶500, Jackson Immunoresearch) and chicken anti-rabbit IgG conjugated with Alexa 488 (1∶100, Molecular Probes). After the final wash with PBS, slides were mounted with Vectashield mounting medium containing DAPI for nuclear staining (Vector Laboratories). Images were acquired using a Zeiss Apotome epifluorescence microscope equipped with a digital camera and Axiovision software. All images were acquired with the same exposure time. Quantification of the IRs was performed by measuring the pixel intensity of the IR-Cy3 fluorescent signal using the Axiovision densitometric function. OB layers (*i.e.*, nerve, glomerular, external plexiform, mitral cell and granular cell layers) were hand-delimited on each image based solely on the DAPI signal. A total of 480 images were acquired and quantified. For each animal, three different zones corresponding to a third of the main OB along the rostro-caudal axis (anterior, intermediate and posterior) were analyzed. In each zone, two frontal sections with left and right OBs were obtained. For each OB frontal section, four images corresponding to the laterodorsal, lateroventral, medioventral and mediodorsal regions of the main OB were acquired. Statistical comparisons were performed using a repeated measures ANOVA with feeding state, zones, regions and layers as factors. A Student-Newman-Keuls post-hoc test was used to complete the analysis when appropriate (Statview software).

## Results

### Validation of the COA paradigm in the two-tube experimental device

**Figure 2:**
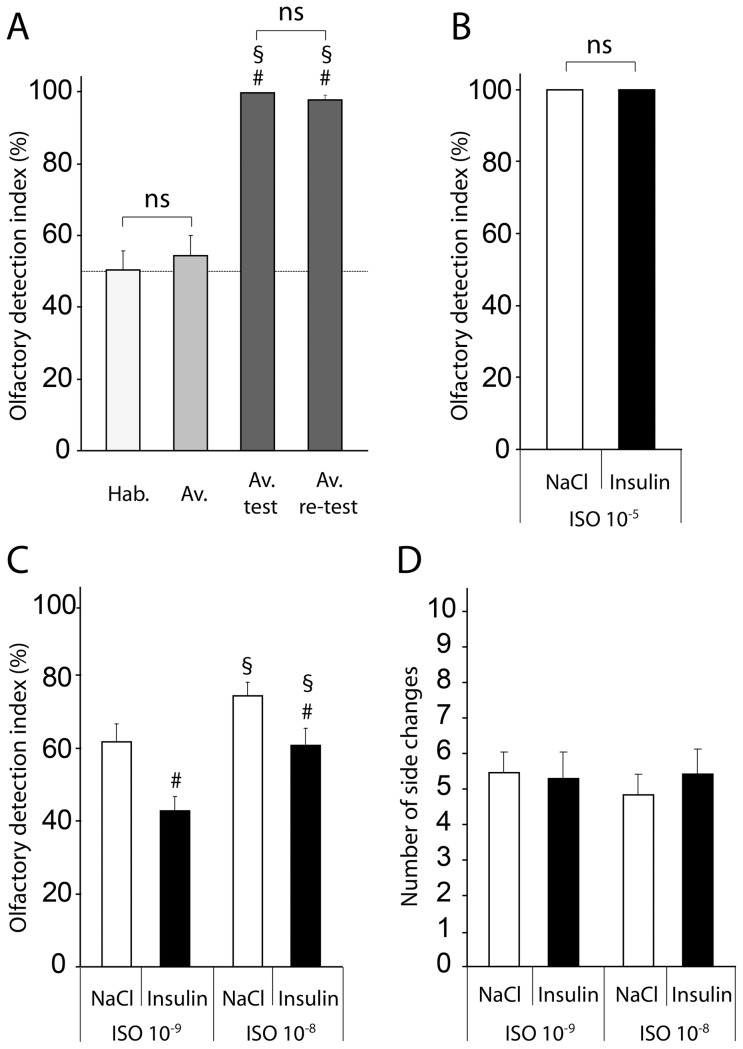
Insulin ICV injection (14 mU) decreases olfactory detection in fasted rats. (*A*) Validation of the COA paradigm in the two-tube experimental device. Before the conditioning, during the last day of habituation (Hab.), both tubes were filled with pure water, and during the first day of aversion acquisition (Av.), both tubes were filled with ISO 10^−5^. A repeated measure ANOVA revealed a significant effect of the aversive conditioning (p<0.0001). For habituation and aversion acquisition, when two identical drinks were proposed, bar graphs represent the olfactory detection index corresponding to the number of licks of the first sampled tube normalized to the total number of licks (mean ± SEM). The mean olfactory detection indexes for habituation and aversion were not statistically different (SNK, p>0.05, ns). After conditioning, rats were given the choice between pure water and ISO 10^−5^ for the aversion test (Aversion test) and re-test (Aversion re-test). Bar graphs represent the olfactory detection index corresponding to the number of licks of the pure-water tube normalized to the total number of licks (mean ± SEM). The mean olfactory detection indexes for the aversion test and re-test were not statistically different (SNK, p>0.05, ns). The mean olfactory detection indexes were significantly different after conditioning (SNK, compared to Hab. # p^1^<0.05, compared to Av. § p^1^<0.05; n = 18). The dashed line represents the chance level (50%). (*B*) Insulin injection before acquisition does not alter aversion retention. Bar graphs represent the olfactory detection index corresponding to the percentage of licks of the pure water tube normalized to the total number of licks (mean ± SEM) when rats were given the choice between pure water and ISO 10^−5^ 1 h after ICV NaCl injection (NaCl) and 1 h after ICV insulin injection (Insulin). The mean olfactory detection indexes were not statistically different (paired t-test, p^1^>0.05, ns; n = 11). (*C*) Insulin ICV injection (14 mU) decreases olfactory detection. Bar graphs represent the olfactory detection index when rats had the choice between pure water and odorized water at ISO 10^−8^ and ISO 10^−9^ 1 h after ICV NaCl injection (NaCl) and 1 h after ICV insulin injection (Insulin). The mean olfactory detection indexes were significantly different for odor dilutions (ANOVA, p^1^<0.005) and ICV treatments (p^1^<0.005; n = 18). Post-hoc comparisons showed that insulin ICV injections significantly decreased the olfactory detection index for both ISO 10^−9^ and ISO 10^−8^ odor dilutions (SNK, compared to NaCl for each odor # p^1^<0.05; n = 18) and that the olfactory detection index is higher for ISO 10^−8^ compared to ISO 10^−9^ for each ICV treatment (SNK, § p^1^<0.05; n = 18) (*D*) Insulin ICV injection (14 mU) does not change locomotor activity. Bar graphs represent the number of side changes in the two-tube experimental device (mean ± SEM) when rats had the choice between pure water, ISO 10^−8^ and ISO 10^−9^ 1 h after ICV NaCl injection (NaCl) and 1 h after ICV insulin injection (Insulin). The mean side changes were not statistically different for odors or ICV treatments (ANOVA, p>0.05, ns; n = 18). ^1^Established using transformed data.

To validate our behavioral paradigm, we measured and compared the number of licks at the first sampled tube normalized to the total number of licks in the experimental device, during a 5 min session for the Habituation, Aversion acquisition, Aversion test and Aversion re-test sessions (Fig. 2A). A repeated measures ANOVA revealed a significant effect of the session (F_(3,17)_ = 61.6, p<0.0001). On the last day of habituation (D3), when the animals had access to two pure-water tubes, they made the same number of licks at both tubes (50.48±5.22%). On the first day of Aversion acquisition (D4) on which the animals had access to two odorized-water tubes at ISO 10^−5^, the rats also made the same number of licks at both tubes (54.39±5.57%). No statistical differences were seen between the last day of habituation and the first day of aversion (SNK, p>0.05), non-significant (ns), indicating that the animals were correctly trained to sample both tubes in the experimental device without any lateralization bias. By contrast, during the Aversion test carried out after the conditioning (on D7), when animals had the choice between a pure-water tube and an odorized-water tube at ISO 10^−5^, the animals drank almost exclusively from the pure-water tube (99.67±0.21%; significantly different from Habituation and Aversion acquisition, SNK, p<0.05), showing that the COA was effective. On the last day of the behavioral experiment (Aversion re-test, on D12), when the animals were again given the choice between a pure-water tube and an ISO 10^−5^ odorized-water tube, animals still drank almost exclusively at the pure-water tube (97.81±1.16%), and this result was not statistically different from the Aversion test result (SNK, p>0.05), indicating that COA was maintained throughout the behavioral experiment.

### ICV insulin injection does not alter COA retention

Central insulin administration is known to modulate several types of memories [27], [36], [41]; [42], [43]. To test whether COA retention was altered by a single and acute ICV insulin injection, on day 15 and 16 (D15 and D16), the rats were given the choice between a pure-water tube and an ISO 10^−5^ odorized-water tube over two consecutive days. We measured the olfactory detection index in the fasted state, either 1 h after a 14 mU insulin ICV injection (D15) or 1 h after an NaCl ICV injection (D16) (Fig. 2B). A paired t-test demonstrated that the olfactory detection index following insulin ICV injection was not statistically different from the olfactory detection index following NaCl ICV injection (p>0.05), indicating that the 14 mU insulin ICV injection had no effect on COA retention.

### ICV insulin injection decreases olfactory detection

In a previous study, we demonstrated that olfactory detection was higher in fasted rats than in satiated ones [15]. To test whether olfactory detection was modulated by ICV insulin injection, following the Aversion test, the conditioned fasted rats were given the choice between pure-water and odorized-water at lower ISO dilutions, ISO 10^−9^ (on D8 and D9) and ISO 10^−8^ (on D10 and D11). For a given odorant dilution, the olfactory detection indexes were measured 1 h after NaCl ICV injection (on D8 and D10) and 1 h after a 14 mU insulin ICV injection (on D9 and D11) (Fig. 2C). A repeated measures ANOVA with odor dilution (ISO 10^−9^ and ISO 10^−8^) and treatment (insulin and NaCl) as factors, demonstrated a significant effect of the odor dilution (F_(1,17)_ = 13.9, p<0.005) and post-hoc comparisons indicated that the olfactory detection indexes were higher for ISO 10^−8^ compared to ISO 10^−9^ (p<0.05). Importantly, a significant effect of treatment was also found (F_(1,17)_ = 13.4, p<0.005) and this effect was independent of the odor dilution effect (F_(1,17)_ = 0.2, p>0.5). Post-hoc comparisons showed that ICV injection of insulin significantly decreased the olfactory detection index for both ISO 10^−9^ (NaCl: 61.81±4.88%, Insulin: 42.74±4.09%, p<0.05) and ISO 10^−8^ (NaCl: 74.38±3.79%, Insulin: 60.83±4.74%, p<0.05) compared to ICV injection of NaCl.

### ICV insulin injection does not alter locomotor activity

Because drastic changes in locomotor activity can affect the sampling behavior in the two-tube experimental device, and could therefore have biased the measure of olfactory detection indexes, we next assessed whether insulin had an effect on locomotor activity in our behavioral set-up. To that end, we compared the mean number of changes of side from the pure water tube to the ISO-odorized water tube during the experimental sessions, 1 h after a 14 mU insulin ICV injection and 1 h after NaCl ICV injection (Fig. 2D). A repeated measures ANOVA with odor dilution (ISO 10^−9^ and ISO 10^−8^) and treatment (insulin and NaCl) as factors demonstrated no significant effect of odor dilution (F_(1,17)_ = 0.074, p>0.7, no significant effect of treatment (F_(1,17)_ = 0.411, p>0.5) and no interaction (F_(1,17)_ = 0.945, p>0.3) on the mean number of side changes, indicating the effect of insulin on olfactory detection was not biased by changes in locomotor activity.

### ICV insulin injection alters sniffing behavior induced by a food odor

It is well-known that in rats, sampling an odor induces sniffing behavior, characterized by an increase in respiratory frequency, [28], [30], [44], [45]. Because insulin has been shown to decrease olfactory sensitivity (data above), we investigated whether insulin is also able to modulate the sniffing behavior in response to food odor. For this, we compared the mean respiratory frequency of animals in three different conditions: fasted animals receiving ICV injection of either NaCl (Fasted-NaCl) or Insulin 14 mU (Fasted-Insulin), and satiated animals receiving ICV injections of NaCl (Satiated-NaCl). In each condition, four 10 sec periods were considered: before (Pre-odor, 10 sec), and during food-odor presentation (30 sec, divided in 3 periods: Odor start = 0–10 sec; Odor middle = 10–20 sec; Odor end = 20–30 sec).

**Figure 3:**
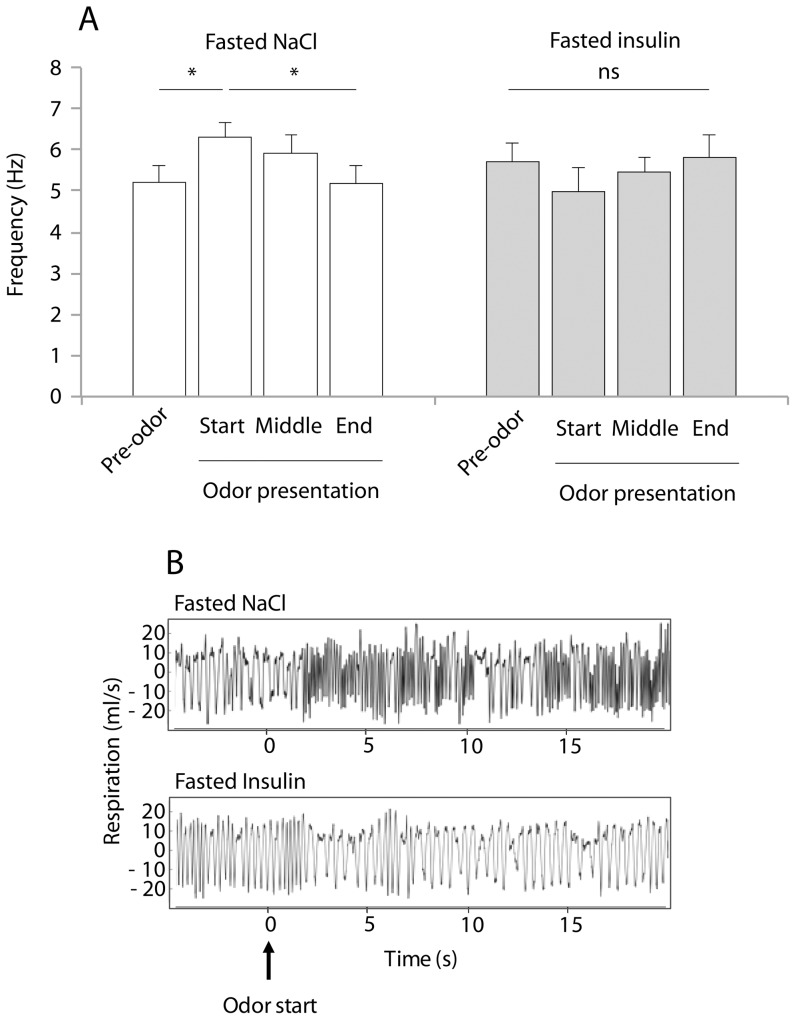
Insulin ICV injection (14 mU) alters olfactory sniffing behavior for a food-odor. *(A)* Bar graphs represent evolution of the mean (± SEM) respiratory frequency during food odor presentation (30 sec), in fasted animals treated with NaCl (n = 10) or insulin (n = 10). Four periods of 10 seconds have been defined: Pre-odor (−10 sec to 0 sec); Odor presentation (start: 0 sec to 10 sec, middle: 10 sec to 20 sec, end: 20 sec to 30 sec). The mean of respiratory frequency of fasted-NaCl animals increased during the presentation of a food odor (Odor presentation, start) compared to Pre-odor period. In Fasted-Insulin rats, no change in respiratory frequency was observed upon odor presentation (Two-way repeated measures ANOVA followed by post-hoc comparisons; * p<0.05 significant differences). *(B)* Individual example of typical time-course of respiration frequency during food odor presentation in two conditions (Fasted rat treated with NaCl or insulin).

When comparing Fasted-NaCl and Fasted-Insulin animals (Fig. 3A), a two-way repeated measures ANOVA (with period and condition as factors) revealed a significant effect for period by condition interaction (F_(3,27)_ = 3.69, p<0.05), but no effect for period or condition alone. In Fasted-NaCl animals, further post-hoc comparisons showed that the presentation of a food odor (Odor start) induced a significant increase in respiratory frequency (p<0.05, compared to Pre-odor period), which progressively decayed until the end of odor presentation (Fig. 3A, left part and Fig. 3B, upper part). In contrast, in Fasted-Insulin rats, no change in respiratory frequency was observed upon odor presentation (Fig. 3A, right part and Fig. 3B, lower part). When comparing Fasted-Insulin animals with Satiated-NaCl animals, the two-way repeated measures ANOVA indicated no effect for period, condition or period by condition interaction (Table 1). Thus, food odor induces sniffing behavior in fasted rats, an effect which was abolished by either physiological satiation or ICV insulin injection.

**Table 1:** Comparison of the mean (± SEM) respiratory frequency in Hz between satiated NaCl (n = 10) and fasted insulin (n = 10) rats during food odor presentation.

		Odor presentation		
	Pre-odor	Start	Middle	End
Fasted insulin	5.395±0.367	4.722±0.595	5. 443±0.382	5.684±0.627
Satiated NaCl	5.587±0.431	5.555±0.429	5.240±0.478	5.201±0.481

Four 10 sec periods were considered: Pre-odor (−10 sec to 0 sec); Odor presentation (start: 0 sec to 10 sec, middle: 10 sec to 20 sec, end: 20 sec to 30 sec). Results are presented for each experimental condition: in the fasted state after 14 mU insulin ICV injection; in the satiated state after NaCl ICV injection. For the four periods, the means of respiratory frequency observed in two experimental conditions were not statistically different. (Two-way repeated measures ANOVA, p>0.05, ns).

### ICV insulin injection does not alter food intake, body weight or peripheral blood glucose levels

Olfactory processing is modulated by the feeding state and insulin-induced hypoglycemia [15], [16], [18], [19], [20], [46], and central insulin administration is well known to decrease food intake and body weight [8], [9], [10], [11], [12], [13]. Therefore, we examined whether insulin infused in the lateral ventricle could act on the hypothalamic nuclei or diffuse in the blood to alter energy homeostasis regulation.

**Figure 4:**
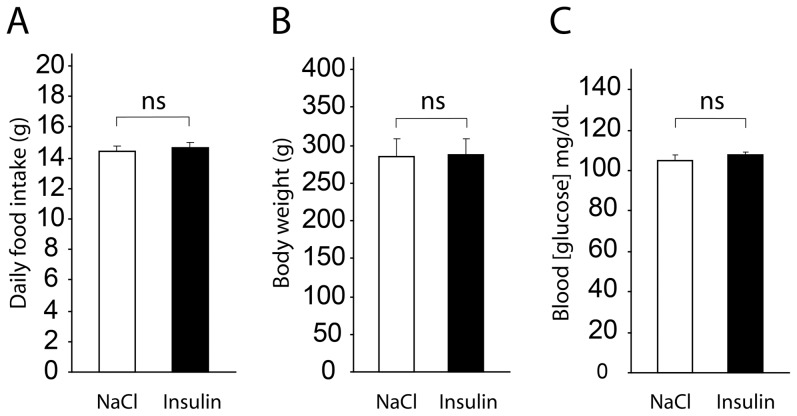
Acute ICV insulin injection does not alter physiological parameters such as food intake, body weight and peripheral blood glucose level. Bar graphs represent food intake *(A)* and body weight *(B)* (mean ± SEM) measured on NaCl ICV injection days (NaCl) and 14 mU insulin ICV injection days (Insulin). The means were not statistically different (paired t-test, p>0.05, ns, n = 18). *(C)* Bar graphs represent the peripheral blood glucose levels (mean ± SEM) measured from tail blood samples 1 h after ICV NaCl injection (NaCl) and 1 h after ICV insulin injection (Insulin). The means were not statistically different (paired t-test, p>0.05, ns; n = 11).

During the olfactory detection experiment (Fig. 1A), we measured and compared the food intake and body weight on NaCl treatment days (D8 and D10) and on 14 mU insulin treatment days (D9 and D11) (Fig. 4A,B). A paired t-test indicated that food intake was not statistically different on ICV insulin injection days (14.70±0.31 g) compared to ICV NaCl injection days (14.43±0.35 g), (p>0.05). Similarly, body weight was not statistically different on ICV insulin injection days (286.28±3.76 g) compared to ICV NaCl injection days (285.40±3.70 g) (p>0.05).

To test the influence of the 14 mU insulin ICV injections on the peripheral blood glucose level, glycemia was measured from tail blood samples (Fig. 4C). A paired t-test indicated that the blood glucose levels were not statistically different on ICV insulin injection days (107.41±1.53 mg/dL) compared to ICV NaCl injection days (105.23±2.22 mg/dL) (p>0.05).

### Feeding state and ICV insulin injection modulate OB insulin levels

**Figure 5:**
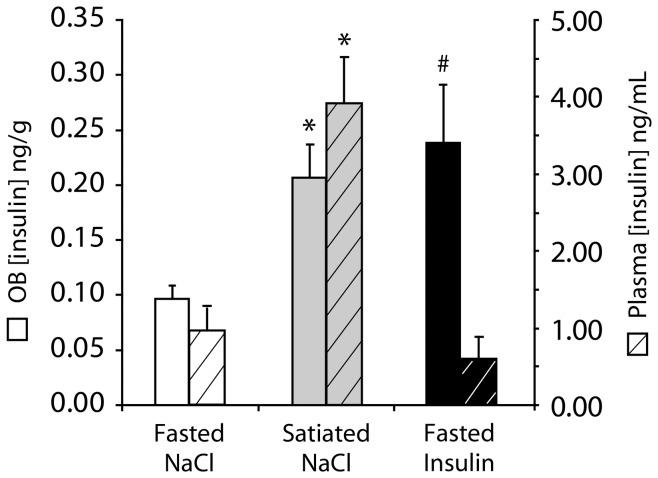
The effects of feeding state and 14 mU insulin ICV injection on blood and OB insulin levels. Bar graphs represent insulin concentrations (mean ± SEM) in the olfactory bulb (OB, full bars, left scale) and plasma (hatched bars, right scale) of fasted (n = 5) and satiated (n = 5) animals 1 h after ICV NaCl injection (Fasted NaCl, Satiated NaCl) or 1 h after ICV insulin injection (Fasted Insulin). The mean OB and plasma insulin levels were statistically different in satiated compared with fasted animals (Mann-Whitney test, * p<0.01), and the mean OB insulin levels were statistically different in fasted animals receiving 14 mU ICV insulin compared with fasted animals receiving NaCl ICV (Mann-Whitney test, # p<0.01).

To examine whether our ICV insulin injection protocol induced an increase of the insulin level in physiological ranges, we measured the OB and plasma insulin levels in both 22 h fasted and satiated animals receiving a NaCl ICV injection (respectively Fasted-NaCl and Satiated-NaCl animals) and in 22 h fasted animals receiving a 14 mU insulin ICV injection (Fasted-Insulin animals) (Fig. 5). In Satiated-NaCl rats, the OB (0.21±0.03 ng/g) and plasma (3.91±0.60 ng/mL) insulin levels were significantly higher than the OB (0.10±0.01 ng/g, Mann-Whitney test, p<0.01) and plasma (0.96±0.29 ng/mL, Mann-Whitney test, p<0.005) insulin levels of Fasted-NaCl animals. Plasma insulin level of Fasted-Insulin animals was not significantly different from that of Fasted-NaCl rats (Mann-Whitney test, p>0.05). By contrast, the OB insulin level of Fasted-Insulin rats (0.24±0.05 ng/g) was significantly higher than that of Fasted-NaCl rats (0.10±0.01 ng/g, Mann-Whitney test, p<0.01), and there was no significant difference between the OB insulin levels of Fasted-Insulin rats and Satiated-NaCl rats (Mann-Whitney test, p>0.05). These results indicate that the ICV injection of insulin 14 mU during our behavioral test selectively increased the OB insulin level of fasted rats to the level observed in satiated animals, without affecting the peripheral blood insulin level.

### Feeding state does not modulate IR mRNA and protein levels in the OB

**Figure 6:**
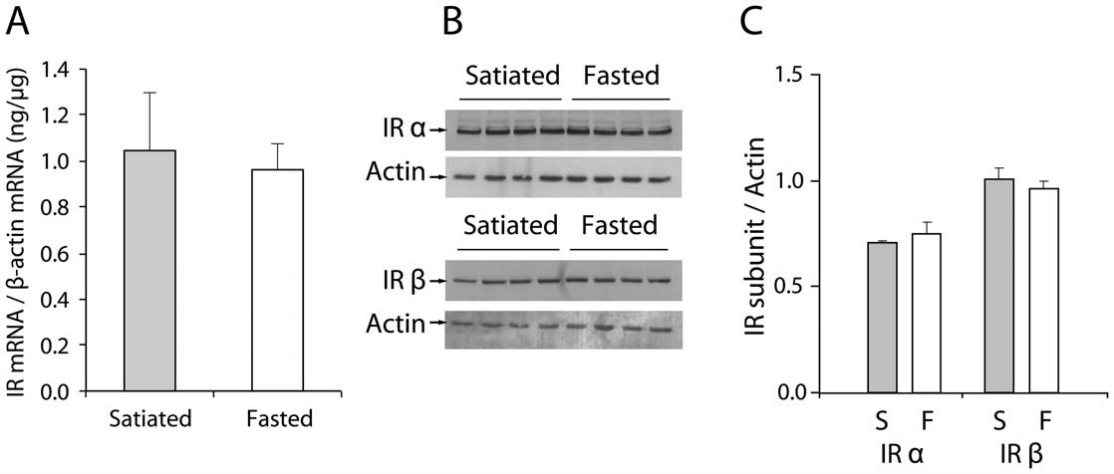
IR mRNA and protein levels in the OB are not modulated by feeding states. *(A)* Bar graphs represent the relative abundance of IR mRNA in the OB of satiated (gray bar; n = 6) and fasted (white bar; n = 6) rats as determined by semi-quantitative RT-PCR and using β-actin mRNA for normalization (mean ± SEM). Representative Western blots *(B)* and quantitative densitometric histograms *(C)* of the IR protein levels in the OB of satiated (gray bar, S; n = 4) and fasted rats (white bar, F; n = 4) (mean ± SEM). Bar graphs represent ratios of the IR subunits (IRα, and IRβ) protein levels after normalization against actin. The means were not statistically different (Mann-Whitney test, p>0.05).

Because a previous study demonstrated that chronic starvation induces a loss of insulin binding sites in the OB [24], we next examined the influence of the feeding state on OB IR content (Fig. 6). A quantitative analysis of IR gene expression showed no significant difference between fasted and satiated states (Fig. 6A: satiated, 1.05±0.25 ng/µg; fasted, 0.96±0.11 ng/µg; Mann-Whitney test, p>0.05). Furthermore, a Western blot analysis of the protein levels of the IR α and β subunits did not reveal any difference in IR protein content between nutritional states (Fig. 6B,C: IRα satiated, 0.71±0.01; IRα fasted, 0.76±0.05; IRβ satiated, 1.01±0.05; IRβ fasted, 0.96±0.03; Mann-Whitney test, p>0.05).

### IR localization and quantification in the main OB

**Figure 7:**
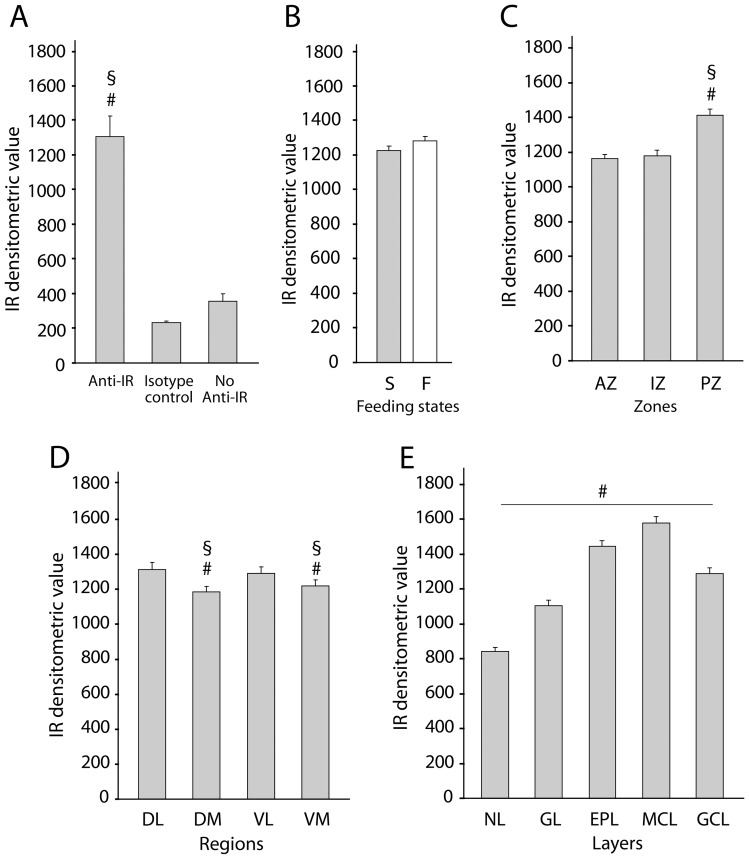
IR quantification in the main OB. Bar graphs represent an arbitrary unit of the IR densitometric value obtained by quantification of the IR-Cy3 immunofluorescent signal on frozen OB sections (mean ± SEM). *(A)* Specificity of the anti-IR antibody. ANOVA of densitometric values revealed significant effect of the antibody used (p<0.001). The mean densitometric value obtained with a monoclonal mouse antibody directed against the β-subunit of the IR (anti-IR), was significantly higher than the mean densitometric value obtained when using either the mouse IgG1 (isotype control) or with the omission of the primary antibody (no anti-IR) (SNK, compared to isotype control # p<0.05, compared to No anti-IR § p^1^<0.05; n = 6). The mean densitometric value obtained with the omission of the primary antibody was significantly higher than the mean densitometric value obtained with the isotype control (SNK, # p<0.05). (B–E) Mean IR densitometric values of the OB sections as a function of feeding states (B), zones (C), regions (D) and layers (E). Repeated measures ANOVA revealed significant effects of zones (ANOVA, p<0.0001), regions (ANOVA, p<0.001) and layers (ANOVA, p<0.0001; n = 10 including 5 fasted rats and 5 satiated rats) but no significant effect of the feeding state (ANOVA, p>0.05). *(B)* Feeding states: The mean IR densitometric values of the OB sections of fasted (F) and satiated (S) animals are similar. *(C)* Zones: The mean IR densitometric value was significantly higher in the caudal posterior zone (PZ) compared to the rostral anterior zone (AZ) and the intermediate zone (IZ) (SNK, compared to AZ # p<0.05, compared to IZ § p<0.05). *(D)* Regions: The mean IR densitometric value was significantly lower in the dorsomedial (DM) and the ventromedial (VM) regions of the main OB compared to the dorsolateral (DL) and ventrolateral (VL) regions (SNK, compared to DL # p<0.05, compared to VL § p<0.05). *(E)* Layers: The mean IR densitometric values measured in the nerve (NL), glomerular (GL), external plexiform (EPL), mitral cell (MCL) and granular cell (GCL) layers of the main OB were all statistically different from each other (SNK, # p<0.05).

**Figure 8:**
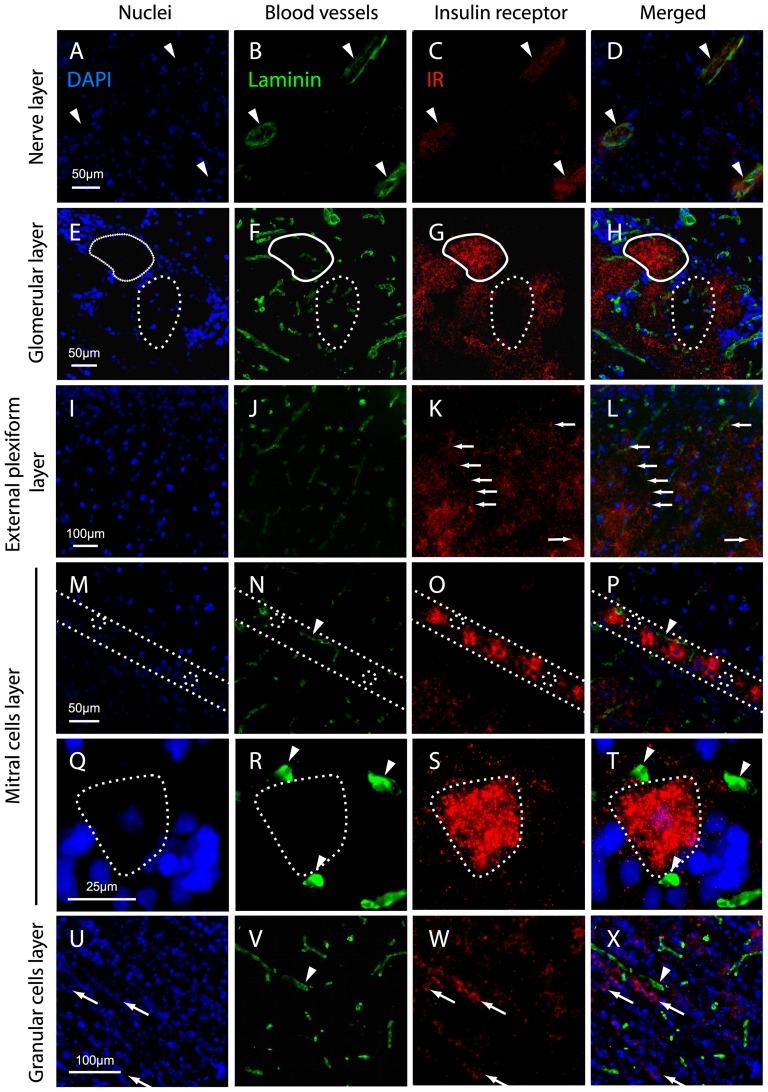
IR localization in the OB layers. Representative images of the various layers of the main OB of adult rats obtained from frontal frozen sections (14 µm) stained with the nuclear marker DAPI *(A, E, I, M, Q, U)* and with double immunostaining of the blood vessel marker laminin *(B, F, J, N, R, V)* and IR *(C, G, K, O, S, W*). The signals were visualized on an epifluorescence microscope using sequential channel scanning with a merged overlay *(D, H, L, P, T, X)*. In the nerve layer *(A–D)*, IR immunostaining is restricted to the endothelium of blood vessels (white arrowheads). In the glomerular layer *(E–H)*, IR immunostaining is abundant is the neuropil of some glomeruli (full line). Some glomeruli are not labeled (dotted line). In the external plexiform layer *(I–L)*, a punctiform IR immunostaining is scattered on labeled fibers (arrows). In the mitral cell layer *(M–P)*, between the dotted lines, IR immunostaining is present in most cell bodies, although some mitral cells are not labeled (dotted circle). The labeled cell bodies are consistently surrounded by blood microvessels (arrowhead). *(Q–T)* Enlargement of a mitral cell body (dotted line) surrounded by three blood microvessels (arrowheads). In the granular cell layer *(U–X)*, IR immunostaining is located in clusters of labeled granular cell bodies (arrows). The labeled clusters are often located close to blood microvessels (arrowhead).

The OB is one of the brain regions that contains the highest level of IRs [21], [22], [23], but the regional distribution of IRs in the OB remains unknown. Using an immunofluorescent densitometric signal, we quantified and studied the regionalization of IRs in the OB network of adult fasted or satiated rats (Fig. 7). To validate the specificity of the IR immunostaining, we selected a random set of 24 OB sections and assigned them to three different immunohistochemistry procedures: i) with a mouse anti-IRβ primary antibody, ii) with an irrelevant antibody (mouse IgG1) for isotype control, or iii) with the omission of the primary antibody (Fig. 7A). ANOVA of densitometric values revealed significant effect of the antibody used (F_(2,21)_ = 34.95, p<0.001). The mean densitometric value obtained when using the anti-IRβ primary antibody (1306.07±117.12) was significantly higher than the mean densitometric value obtained when using either the isotype control (232.22±9.53, SNK, p<0.0001) or no primary antibody (356.52±38.90, SNK, p<0.0001). Additionally, the omission of the primary antibody resulted in significantly more background than the isotype control procedure (SNK, p<0.05). A repeated measures ANOVA of densitometric values with feeding state, OB zone, region and layer as factors revealed no effect of feeding state (F_(1,8)_ = 0.076, p>0.7, Fig. 7B), but showed a significant effect of zone (F_(2,16)_ = 22.41, p<0.0001, Fig. 7C), region (F_(3,24)_ = 8.30, p<0.001 Fig. 7D) and layer (F_(4,32)_ = 183.37, p<0.0001 Fig. 7E). Concerning the OB zones (Fig. 7C), the SNK post-hoc test revealed that the posterior zone had a significantly higher mean densitometric value (1412.64±34.61) than the other two zones (anterior, 1164.42±22.66; intermediate, 1179.23±31.70), which were not significantly different from each other. Concerning the OB regions (Fig. 7D), the dorsolateral region (1314.59±37.55) and the ventrolateral region (1290.19±39.72) had a greater mean densitometric value than the dorsomedial (1185.52±30.39) and the ventromedial (1218.09±34.61) regions. Finally, concerning the OB layers (Fig. 7E), the mean densitometric value of each layer of the OB was significantly different from the mean densitometric values of all of the other layers (p<0.0001). The mitral cell layer showed the highest densitometric value (1577.54±40.18), and the nerve layer displayed the lowest densitometric value (841.69±24.78). Between these, the external plexiform (1446.06±33.91), the granular cell (1288.91±34.23) and the glomerular (1106.28±24.41) layers demonstrated intermediate decreasing mean densitometric values. Representative images of IR immunostaining among the OB layers are presented in Fig. 8.

Since the insulin secreted by the pancreas crosses the blood-brain barrier to reach the CNS [4], [5], [6], [7], we analyzed the relationship between IR localization and OB microvasculature. For this, double labeling of laminin and IR was performed on the same sections. In the nerve layer (Fig. 8, A–D), the IR immunoreactivity was weak and was restricted to the endothelium of large OB blood vessels. In the glomerular layer (Fig. 8, E–H), the IR immunoreactivity was mainly restricted to the glomerular neuropil and was independent of the rich microvasculature irrigating each glomerulus. Some glomeruli were not labeled, and the soma of periglomerular cells surrounding each glomerulus did not present any IR signal. A strong and diffused IR immunoreactivity was observed in the external plexiform layer (Fig. 8, I–L). The IR immunoreactivity was independent of the microvasculature, and some scattered labeled fibers either crossing the external plexiform layer close to the mitral cell layer or at the entrance of the glomeruli, were observed. In the mitral cell layer (Fig. 8, M–T), most cell bodies were intensely labeled, though some mitral cells showed no immunoreactivity. Interestingly, labeled mitral cells were consistently surrounded by microvessels. In the granular cell layer (Fig. 8, U–X), the IR immunoreactivity was patterned in clusters of labeled granular cell bodies, but some clusters had no detectable immunoreactivity. Labeled granular cell clusters were consistently located close to an OB microvessel.

## Discussion

The present study provides new information on the role of insulin on olfactory behaviors and olfactory processing at the OB level. We demonstrated that insulin ICV injection in fasted adult rats decreased olfactory detection of a learned aversive odor and abolished sniffing behavior during food odor presentation. These effects were not biased by changes in locomotor activity or an alteration of odor memory retrieval. Furthermore, insulin ICV injection in fasted rats increased the OB insulin level to the physiological level found in satiated rats without affecting peripheral insulin level, blood glucose level, daily food intake or body weight. Moreover, we quantified IRs distribution and showed that IRs are preferentially expressed in the caudal and lateral parts of the main OB, with the highest density of IRs in the cell bodies of the main projection neurons, the mitral cells, in close vicinity of the OB microvessels. Together, these data indicate that insulin which in physiological conditions, crosses the blood-brain barrier to reach the CNS, may modulate olfactory information processing in the OB to decrease olfactory sensitivity and alter food odor-induced sniffing behavior.

### ICV insulin injection decreases olfactory detection

In a previous study, we reported that fasting increased whereas satiation decreased olfactory sensitivity in the COA paradigm [15]. The present data show that an ICV injection of insulin in fasted animals induced a decrease in olfactory detection of a learned aversive odor, thus mimicking the effects of satiation. Because central insulin administration is known to modulate several types of memories [27], [36], [41], [42], [43], we assessed the effect of ICV insulin injection on COA retention. We found that ICV insulin injection carried out 1 h before the COA retention test did not alter the animals' performances. Therefore the decreased olfactory detection observed following the ICV injection of insulin cannot be ascribed to the disruption of mnesic processes but rather to the alteration of sensory processes. Consistent with our results, a growing body of evidence indicates that insulin modulates olfactory processing. In humans, a short report recently indicated that subjects submitted to a hyperinsulinemic-euglycemic clamp demonstrate a decrease of olfactory detection [47]. In parallel, we and others recently reported that fasting and orexigenic molecules such as orexin A and ghrelin increase olfactory sensitivity, while satiation and anorectic hormones such as insulin and leptin have the opposite effect (for review see [48]).

Moreover the present study shows that ICV insulin injection alters respiratory response to a familiar food odor. Respiration and olfaction are intimately linked since in mammals, odor sampling occurs through inhalation of air through the nose. Sampling a novel odorant results in reliable induction of high-frequency (6–10 Hz) sniffing [28], [44], [45], [49], [50]. The strong modulation of sniffing behavior during odor sampling has led to the idea that sniffing plays a critical role in odor information processing [29], [51], [52], [53], [54], [55], and it is well established, that a high nostril flow rate is associated with enhanced odorant detection [56], [57]. In the present study we showed that in fasted animals, presentation of the familiar food odor induced an increase in sniffing frequency 1–2 sec after the odor onset. Studies in the literature showing that changes in sniff frequency generally occur in the 200–300 msec range have used either a nose poke-based odor-sampling paradigm [58], [28], [45], [59] or a head-fixed paradigm [60], [61]. In both paradigms the nose of the rat is close to the odor source, thus explaining the very short latency of the observed changes in sniff frequency. In our study, the odorant is delivered from the top of the plethysmograh and the 1–2 sec delay before the changes reflects the delay for the odorant to diffuse throughout the chamber and be detected by the animal. Previous studies investigating freely expressed exploratory sniffing in unrestrained rodents have reported similar latencies and durations in sniff frequency changes [30], [28].

Very few studies have investigated the effect of fasting and orexigenic molecules on odor-induced sniffing behavior. In the present study we showed that the satiety hormone insulin, like satiation, abolished sniffing behavior in response to food odor presentation. Interestingly, consistent our data, a recent study revealed that ghrelin, a fasting hormone, increases sniffing behavior both in rats and humans [62]. Altogether, these behavioral results indicate that food-intake related molecules modulate both sniffing behavior and olfactory detection.

### ICV insulin injection has no effect on food consumption or body weight

In this study we show for the first time that an insulin injection which yields a physiological increase of insulin level in the OB, decreases the sniffing behavior in response to food odor. This effect could result from either a decrease in food oriented motivation, or a decrease in olfactory sensory sensitivity. The former hypothesis seems unlikely since in the present study, insulin injection had no effect on food consumption or body weight. This contrasts with many reports indicating that central insulin administration decreases food intake [8], [9], [10], [11], [13]. This can rely first, on the fact that we made acute injections into the lateral ventricle to preferentially target the OB, whereas in most reports, insulin is injected chronically into the third ventricle surrounded by the hypothalamus. Indeed, a previous report indicates that an acute infusion of insulin into the lateral ventricle has no effect on food intake and body weight [63]. Second, our rats were submitted to a chronic food-restriction schedule, and insulin does not modulate food intake in food-deprived animals [10], [12]. Therefore we suggest that the decrease in sniffing behavior in response to food odor following insulin injection rather reflects the decrease in olfactory sensory detection.

### ICV insulin injection mimics a physiological increase of insulin

In animals, IRs were shown to be expressed by olfactory sensory neurons and sustentacular cells of the olfactory epithelium[27], [64], and insulin decreases the evoked response of olfactory sensory neurons to neutral odors [64], [65]. In the OB, the voltage-dependent potassium channel Kv1.3, a downstream target of IR kinase activity, is highly expressed [66], [67], [68] and Kv1.3 −/− mice have a “supersmeller” phenotype [69]. In normal mice, repeated intranasal insulin delivery induces Kv1.3 phosphorylation and facilitates olfactory discrimination [27]. However, no changes in olfactory detection were reported. By contrast, we demonstrated here that insulin ICV injection decreases olfactory detection. Based on our ELISA measurements and the intranasal delivery method's efficiency reported by Marks et al. [27], one can estimate that the amount of insulin effectively reaching the OB through ICV injection is ∼10 times higher than through the intranasal delivery route, which may underlie this apparent discrepancy. We showed that the feeding state modulates OB insulin content, the OB insulin level being higher in satiated than in fasted rats. Importantly, our ICV injection protocol yielded a physiological increase of insulin, by elevating the OB level of fasted rats to that of satiated rats. We also found that changes in OB insulin level correlated with changes in plasma insulin level, suggesting that the OB is highly sensitive to fluctuations in circulating insulin levels. This result is consistent with the general agreement that most insulin entering the brain is produced by the pancreas and transported across brain capillaries by a receptor-mediated saturable transporter [6], [7], [70], [71]. The rate of insulin entrance into the brain is regulated by several physiological factors, including the feeding state [70], [72]. When animals are fasted, the ability of insulin to cross the blood-brain barrier is reduced, leading to a positive correlation between blood and cerebrospinal fluid insulin levels [73].

### Distribution of insulin receptors within the olfactory bulb

IRs are expressed in several olfactory brain regions, including the anterior olfactory nucleus, piriform cortex, olfactory tubercule and OB, the latter being consistently reported to be the brain region containing the highest level of IRs [21], [22], [23]. Although one report suggested that OB insulin binding is modulated by the feeding state and reduced by chronic fasting [24], a more recent study indicated that in brain regions involved in energy homeostasis regulation, nutrient availability does not modulate IR levels [74]. Consistent with the latter, we demonstrated here that the OB IR expression is not modulated by the feeding state. We found that the mitral cell layer had the highest density of IRs, followed by the external plexiform, the granular cell and the glomerular cell layers. Among the various neurons of the OB network, IRs were abundantly expressed in mitral and granular cell bodies, whereas no staining was observed in periglomerular cells. The localization of mitral and granular cells expressing IRs close to blood vessels renders them particularly sensitive to plasma insulin level fluctuations. Mitral cells are glutamatergic OB projection neurons, and granular cells are GABAergic interneurons known to modulate mitral cells activity through dendrodendritic synapses in the external plexiform layer [75]. Interestingly, IRs were found in the external plexiform layer, where dendrodendritic synapses have been demonstrated to be major insulin-receptive sites [76]. IRs were also found in some glomerular neuropils, an OB functional unit in which mitral cells receive direct excitatory glutamatergic input from olfactory sensory neurons and inhibitory input from periglomerular GABAergic interneurons [77]. Together, these data indicate that insulin activity in the main projection neurons, in interneurons and in synaptic contact layers may participate in the modulation of synaptic transmission of odor-related stimuli. Our quantitative immunofluorescence procedure demonstrated a regionalization of the OB IRs along the rostro-caudal and latero-medial axes of the OB. The posterior and lateral regions of the main OB presented the highest amount of IRs. The mammalian OB exhibits a spatial organization of odor maps in relation to the molecular features of odorants [31], [32], [78] and the olfactory stimulation routes [79]. Thus, the posterior and lateral OB regions are preferentially activated by odorants with high water solubility and by retronasal presentations of odorants [78], [79]. During food consumption, because of warming and mastication, the odorant concentration reaching the nasal cavity through retronasal stimulation is much higher than through orthonasal stimulation [80]. In humans, retronasal olfactory stimulation influences satiation and affects food intake behaviors [81], [82], [83]. Therefore, the IR regionalization in the posterior and lateral regions of the OB may be linked to the processing of food intake-related odors.

In the present study we demonstrated that the expression and distribution of IRs in the OB are not changed by the feeding state. However, similarly to the satiated state, following an ICV insulin administration, the insulin concentration increases in the OB and insulin reaches its receptors located in the OB network, ultimately leading to a decrease of olfactory detection and a suppression of food odor-induced sniffing behavior. Here, we provided some robust arguments suggesting that in addition to their classical role in energy homeostasis regulation, metabolic signals such as insulin can modulate food intake-related odor processing, via their action within the olfactory bulb.
